# Histamine, histamine receptors, and neuropathic pain relief

**DOI:** 10.1111/bph.14696

**Published:** 2019-06-07

**Authors:** Ilona Obara, Vsevolod Telezhkin, Ibrahim Alrashdi, Paul L. Chazot

**Affiliations:** ^1^ School of Pharmacy Newcastle University Newcastle upon Tyne UK; ^2^ Institute of Neuroscience Newcastle University Newcastle upon Tyne UK; ^3^ School of Dental Sciences Newcastle University Newcastle upon Tyne UK; ^4^ Department of Biosciences Durham University Durham UK

## Abstract

Histamine, acting via distinct histamine H_1_, H_2_, H_3_, and H_4_ receptors, regulates various physiological and pathological processes, including pain. In the last two decades, there has been a particular increase in evidence to support the involvement of H_3_ receptor and H_4_ receptor in the modulation of neuropathic pain, which remains challenging in terms of management. However, recent data show contrasting effects on neuropathic pain due to multiple factors that determine the pharmacological responses of histamine receptors and their underlying signal transduction properties (e.g., localization on either the presynaptic or postsynaptic neuronal membranes). This review summarizes the most recent findings on the role of histamine and the effects mediated by the four histamine receptors in response to the various stimuli associated with and promoting neuropathic pain. We particularly focus on mechanisms underlying histamine‐mediated analgesia, as we aim to clarify the analgesic potential of histamine receptor ligands in neuropathic pain.

**Linked Articles:**

This article is part of a themed section on New Uses for 21st Century. To view the other articles in this section visit http://onlinelibrary.wiley.com/doi/10.1111/bph.v177.3/issuetoc

**Abbreviations**
CaM: calmodulin
DRG: dorsal root ganglion
GSK3β: glycogen synthase kinase 3β
IP_3_: inositol triphosphate
KO: knockout
LC: locus coeruleus
PIP_2_: phosphatidylinositol 4,5‐bisphosphate
PNS: peripheral nervous system
SP: substance P

## 1 NEUROPATHIC PAIN AND ITS TREATMENT

Neuropathic pain was first defined by the International Association for the Study of Pain as “pain initiated or caused by a primary lesion or dysfunction in the nervous system” (Merskey & Bogduk, 1994). Fourteen years later, Treede et al. (2008) revised this definition and redefined it as “pain arising as a direct consequence of a lesion or disease affecting the somatosensory system.” In the revised definition of neuropathic pain, two terms have received particular attention. First, the term “disease” which refers to all types of abnormal conditions including inflammation, autoimmune syndromes, and ion channel disorders (channelopathies) replaced the term “dysfunction.” Second, to avoid misdiagnosis of neuropathic pain as another type of pain originating from the nervous system, such as spasticity and rigidity of the muscles and bone (e.g., musculoskeletal pain), the term “nervous system” was replaced by the term “somatosensory system” (Finnerup et al., 2016). This revised definition of neuropathic pain describes the nature of this condition more precisely and is, therefore, now widely accepted and approved by the Neuropathic Pain Special Interest Group of the International Association for the Study of Pain. Neuropathic pain can be divided into two subtypes, peripheral or central, based on the anatomical location of the lesion or the disease, within the peripheral nervous system (PNS) or central nervous system (CNS), respectively.

It is estimated that the worldwide prevalence rate of neuropathic pain in the general population lies between 7% and 10%; however, this figure differs for different countries (van Hecke, Austin, Khan, Smith, & Torrance, 2014). The highest prevalence rates for neuropathic pain were recorded in Canada (17.9%) and in the United States (9.8–12.4%; VanDenKerkhof et al., 2016; Yawn et al., 2009), while a relatively low prevalence rate was noted in Austria and Netherlands (3.3% and 1%, respectively; Bouhassira, Lanteri‐Minet, Attal, Laurent, & Touboul, 2008; Gustorff et al., 2008; Harifi et al., 2013). In the United Kingdom, France, and Brazil, it is reported that 7–10% of chronic pain sufferers have been affected by neuropathic pain (Fayaz, Croft, Langford, Donaldson, & Jones, 2016). As a consequence of extended life expectancy, it is predicted that the worldwide prevalence rate of neuropathic pain is likely to increase further, because this type of chronic pain occurs with many common age‐related diseases. Neuropathic pain is triggered by a lesion within the somatosensory system, trauma, or to toxic effects of certain medications (Colloca et al., 2017; Yu et al., 2013). Pathological conditions that are responsible for the development of neuropathic pain through injury include metabolic diseases (e.g., diabetic neuropathy), infection (e.g., postherpetic neuralgia), vascular disease (e.g., stroke), trauma (e.g., orofacial neuropathy), and cancer (Campbell & Meyer, 2006). Neuropathic pain is a complex condition that can either be constant or periodic and presents with a range of different symptoms. These symptoms can increase throughout the day with clinically relevant morning–evening differences and can be affected by gender and underlying aetiology (Gilron, Bailey, & Vandenkerkhof, 2013). Sufferers of neuropathic pain have ongoing, spontaneous pain that has a significant negative impact on quality of life and daily functioning, including physical, emotional, and social well‐being (Jensen, Chodroff, & Dworkin, 2007).

The mechanisms underlying neuropathic pain are complex and multidimensional. Numerous pathophysiological and biochemical changes cause morphological and functional adaptations in the nervous system, including an increase in excitatory neurotransmitters and neuropeptides, for example, http://www.guidetopharmacology.org/GRAC/LigandDisplayForward?ligandId=1204, http://www.guidetopharmacology.org/GRAC/LigandDisplayForward?ligandId=649, 5‐HT, and glutamate, which leads to hyperexcitability (Baron, Binder, & Wasner, 2010). Likewise, injured peripheral nerve fibres give rise to an intense and prolonged input of ectopic activity to the CNS and may induce secondary changes to the excitability of the spinal cord dorsal horn neurons (Colloca et al., 2017; Ossipov & Porreca, 2005). These morphological changes and functional adaptations lead to abnormal sensory signs in patients with neuropathic pain presented as, for example, allodynia (pain due to a stimulus that does not normally activate the nociceptive system), hyperalgesia (an increased response to a stimulus that is normally painful), or sensory loss (hypoesthesia). Clinically, neuropathic pain manifests as evoked pain and presents in many ways such as burning, tingling, prickling, shooting, electric shock‐like, jabbing, squeezing, spasm, or cold (Rice, Finnerup, Kemp, Currie, & Baron, 2018).

Animal models of neuropathic pain are essential in understanding the plethora of mechanisms that may drive neuropathic pain, allowing the field to identify potential therapeutic targets for the effective management of this condition. Extensive research in the pain field has developed and characterized a wide variety of animal models of neuropathic pain. The four most commonly used models, also adopted in some studies discussed in this review, are chronic constriction injury (Bennett & Xie, 1988), partial sciatic ligation (Seltzer, Dubner, & Shir, 1990), spinal nerve ligation (Kim & Chung, 1992), and spared nerve injury (Decosterd & Woolf, 2000) models. These models aim to simulate some of the clinical features of neuropathic pain, in the preclinical setting (e.g., allodynia and hyperalgesia), because a large proportion of peripheral neuropathic pain models, which are currently used in research, share alterations in hind‐limb cutaneous sensory thresholds following partial injury of a peripheral (usually sciatic) nerve. This is associated with the development of neuropathic pain symptoms, such as hyperalgesia and allodynia (Ma et al., 2003; Wall et al., 1979). There are also limitations associated with the use of animal models of neuropathic pain. These limitations are linked to challenges associated with (a) reliable and objective measures of behavioural responses to noxious stimuli, since animals cannot self‐report and the experimenter can be biased, (b) appropriateness of the outcome measures, for example, sleep disturbance to reflect spontaneous pain, and (c) complexity of mechanisms underlying the development of neuropathic pain and their relevance to humans (Colleoni & Sacerdote, 2010).

Despite advances in the understanding of the underlying causes and mechanisms leading to the development and maintenance of neuropathic pain, 40% of Europeans who suffer from chronic pain did not achieve satisfactory pain control (Breivik, Collett, Ventafridda, Cohen, & Gallacher, 2006), and to date, no medication has shown long‐term efficacy and tolerability for neuropathic pain conditions. A significant contributing factor to these limited therapeutic strategies is that neuropathic pain has different aetiology and pathophysiology to any other type of chronic pain, making the management of this type of chronic pain particularly difficult and challenging (Finnerup et al., 2015). Consequently, there is a pressing need for the identification of new therapeutic strategies to improve management of neuropathic pain that will directly improve the outcome for pain sufferers.

The histamine system has been a target for multiple therapeutic interventions. Recently, growing evidence has supported the use of selective ligands of histamine http://www.guidetopharmacology.org/GRAC/ObjectDisplayForward?objectId=264 and http://www.guidetopharmacology.org/GRAC/ObjectDisplayForward?objectId=265 for the treatment of neuropathic pain (Bhowmik, Khanam, & Vohora, 2012; Chaumette et al., 2018; Popiolek‐Barczyk et al., 2018; Sanna, Mello, Masini, & Galeotti, 2018). Approval in the European Union for the use of http://www.guidetopharmacology.org/GRAC/LigandDisplayForward?ligandId=8924 (Wakix™), an antagonist/inverse agonist of H_3_ receptors for the treatment of narcolepsy (Kollb‐Sielecka et al., 2017), presents an opportunity to explore its clinical use for other conditions. Consequently, it seems an appropriate time to reconsider the histamine system as a therapeutic target for the management of neuropathic pain. This review aims to summarize the most recent findings on the role of histamine and its effects, mediated by different subtypes of histamine receptors, on neuropathic pain, with particular regard to the mechanisms underlying histamine‐mediated analgesia.

## 2 HISTAMINE, HISTAMINE RECEPTORS, AND PAIN

### 2.1 Histamine and pain

Histamine (2‐(4‐imidazolyl)‐ethylamine), one of the most extensively studied amino acid‐derived neurotransmitters in the CNS and PNS, is involved in various physiological and pathological processes, including sleep‐waking cycle, homeostasis, synaptic plasticity, and learning (Panula et al., 2015; Parsons & Ganellin, 2006; Pini, Obara, Battell, Chazot, & Rosa, 2016). Histamine is synthesized from the amino acid http://www.guidetopharmacology.org/GRAC/LigandDisplayForward?ligandId=3310 through oxidative decarboxylation via catalysis with the histidine decarboxylase enzyme (Bodmer, Imark, & Kneubuhl, 1999; Haas, Sergeeva, & Selbach, 2008) and is arguably the most pleiotropic molecule in the human and animal body, being present in many cell types (Lindskog, 2017). Histamine is released by neuronal and non‐neuronal sources and is responsible for many physiological processes, including the contraction of smooth muscles of the lungs, uterus, and intestine, secretion of gastric acid in the stomach, and vasodilation, and modulation of heart rate and contractility (Parsons & Ganellin, 2006). Histamine also functions as a neurotransmitter, within the nervous system, regulating a variety of body functions, such as temperature control, memory, wakefulness, and pain sensation (Panula & Nuutinen, 2013).

Histamine is a key mediator in the processing of nociceptive information, acting in an antinociceptive manner in the CNS while, conversely, in a nociceptive manner in the PNS (Khalilzadeh, Azarpey, Hazrati, & Vafaei Saiah, 2018). In the PNS, histamine is released in response to tissue injury/damage, and, through the sensitization of polymodal nociceptors resulting in increased firing rates, it contributes to the generation of pain hypersensitivity. In neuropathic pain, histamine released in the periphery by mast cells has been shown to play an important role in the development of hypersensitivity following nerve injury. This pathological process is associated with recruitment of macrophages and neutrophils, and as histamine is a powerful chemoattractant of mast cells, it regulates this recruitment (Smith, Haskelberg, Tracey, & Moalem‐Taylor, 2007; Zuo, Perkins, Tracey, & Geczy, 2003). Interestingly, it was also observed that peripherally acting histamine could interact with mechanisms underlying pruritus (itch) and pain. Findings suggest that low concentrations of histamine, acting on sensory neurons, produce pruritus with a high concentration leading to pain (Baron, Schwarz, Kleinert, Schattschneider, & Wasner, 2001; Hough & Rice, 2011; LaMotte, Simone, Baumann, Shain, & Alreja, 1987; Parsons & Ganellin, 2006; Pini et al., 2016; Simone, Alreja, & LaMotte, 1991). There is also evidence to show that histamine‐induced itch can convert into pain associated with neuropathic hyperalgesia (Baron et al., 2001). Indeed, multiple itch pathways were identified indicating the presence of distinct itch‐generating types of neuron, one responsible for transmitting itch sensation and the other, ultimately, for transmitting pain (Usoskin et al., 2015). In contrast to histamine activity in PNS, multiple behavioural studies have shown that histamine injected directly into the various brain areas (e.g., somatosensory cortex or hippocampus) attenuated pain (Erfanparast, Tamaddonfard, Farshid, & Khalilzadeh, 2010; Tamaddonfard & Hamzeh‐Gooshchi, 2014).

Histamine exerts its effects via four distinct GPCR subtypes: http://www.guidetopharmacology.org/GRAC/ObjectDisplayForward?objectId=262, http://www.guidetopharmacology.org/GRAC/ObjectDisplayForward?objectId=263, H_3_, and H_4_ receptors. These receptors differ in their pharmacology and signal transduction properties (Panula et al., 2015; Parsons & Ganellin, 2006; Simons & Simons, 2011). Thus, histamine has differential effects on neuropathic pain dependent upon the histamine receptor subtype it is bound to. As mentioned previously, this review aims to summarize histamine‐mediated effects on neuropathic pain. Therefore, the next sections of this review focus on mechanisms underlying histamine‐mediated analgesia.

### 2.2 Histamine receptors and pain

Excitatory histamine receptor signalling in nociceptive pathways is associated with increased pain symptoms (Gangadharan & Kuner, 2013; Mobarakeh et al., 2000), whereas inhibition of histamine receptor signalling predominantly causes neuroprotective and antinociceptive effects (Bhowmik et al., 2012; Chazot & Care, 2005; Popiolek‐Barczyk et al., 2018). Different subtypes of histamine receptors are expressed in both presynaptic and postsynaptic neuronal membranes (Brown, Stevens, & Haas, 2001; Parsons & Ganellin, 2006; Zhang et al., 2013). Presynaptic histamine receptors function as autoreceptors or heteroreceptors providing either positive or negative feedback regulation of neurotransmitter release from the axon terminals into the synaptic cleft (Nieto‐Alamilla, Marquez‐Gomez, Garcia‐Galvez, Morales‐Figueroa, & Arias‐Montano, 2016). It could be concluded that the resultant excitatory or inhibitory physiological effect of histamine receptors depends on the action of the neurotransmitter and the subsequent downstream cascade.

Specificity of localization of histamine receptors in different parts of the nervous system, on either presynaptic or postsynaptic membranes, is determined by their physiological relevance (Parsons & Ganellin, 2006). Among the four subtypes of histamine receptors, H_1_ and H_2_ receptors are predominantly identified postsynaptically (Brown et al., 2001; Connelly et al., 2009; Zhang et al., 2013), with the location of the H_4_ receptor requiring further investigation (Connelly et al., 2009). Cross‐desensitization and agonist‐induced heterodimerization of H_1_ and H_2_ receptors (Alonso et al., 2013) may suggest a possible partnership between histamine receptors. Expression of H_3_ receptors was initially reported as exclusively presynaptic in the rat cerebral cortex (Arrang, Garbarg, & Schwartz, 1983; Clark & Hill, 1996), while postsynaptic expression of H_3_ receptors could not be completely excluded (Nieto‐Alamilla et al., 2016). H_3_ receptors are predominantly expressed in neurons and, together with H_4_ receptors, have higher affinity (nM range) for histamine than H_1_ and H_2_ receptors (μM range; Parsons & Ganellin, 2006). Expression of H_3_ and H_4_ receptors on the opposite sides of the synaptic cleft may contribute to their effects in neuropathic pain, although the neuronal topology of the H_4_ receptor still remains controversial. The use of selective ligands for histamine receptors has led to a better understanding of the physiological and pathophysiological roles of these receptors. The next section summarizes the effects produced by histamine receptor ligands on neuropathic pain.

Besides their presynaptic or postsynaptic localization, the physiological effects of histamine receptors are, to a great extent, determined by the type of guanine nucleotide‐binding proteins (G‐proteins) to which they are coupled (Leung & Wong, 2017). The difference in underlying signalling pathways may directly determine the effect on pain perception produced by selective ligands, even when they act at the same histamine receptor, as described below.

#### 2.2.1 H_1_ receptor

**Figure 1:**
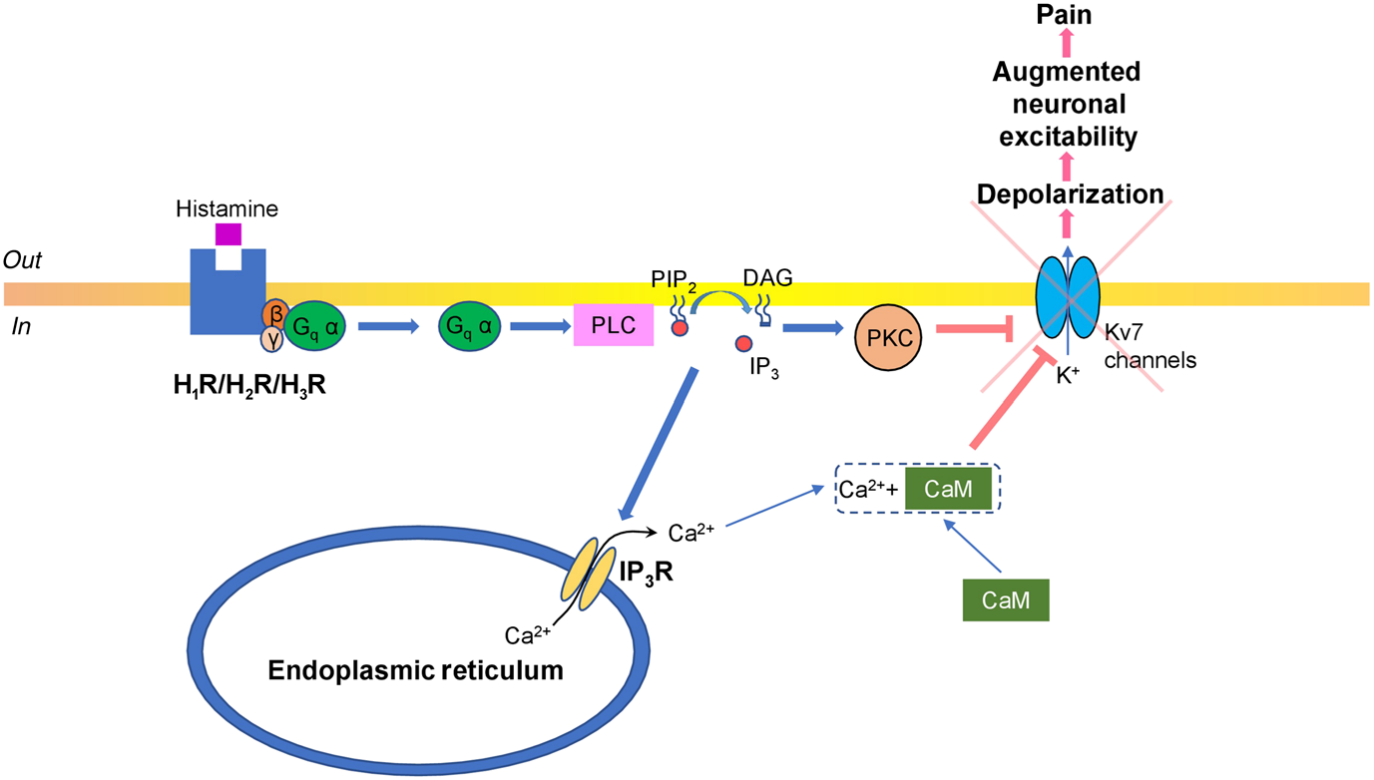
Diagram illustrating histamine receptor signalling—G_q_ pathway. Histamine binds to histamine receptors (H_1_, H_2_, or H_3_ receptor subtypes) that are coupled with the G_q_‐type protein. G_q_ α subunit activates PLC which hydrolyses phosphatidylinositol 4,5‐bisphosphate (PIP_2_), subsequently producing DAG, that remains in the inner leaflet of the plasma membrane activating PKC, and water soluble inositol triphosphate (IP_3_), which binds to http://www.guidetopharmacology.org/GRAC/FamilyDisplayForward?familyId=123 (IP_3_R) and stimulates Ca^2+^ release from endoplasmic reticulum. This intracellular Ca^2+^ forms a complex with calmodulin (CaM) Ca^2+^CaM and induces PKC‐dependent phosphorylation. This suppresses the activity of potassium voltage‐gated channels type 7 (K_v_7 channels), which depolarizes the neurons, and leads to the augmentation of neuronal excitability, which manifests as increased pain symptoms

H_1_ receptors are excitatory receptors, which couple with G_q_‐type proteins, leading to downstream activation of http://www.guidetopharmacology.org/GRAC/FamilyDisplayForward?familyId=274 and hydrolysis of http://www.guidetopharmacology.org/GRAC/LigandDisplayForward?ligandId=2387 (PIP_2_) to produce DAG and http://www.guidetopharmacology.org/GRAC/LigandDisplayForward?ligandId=4222 (IP_3_). DAG subsequently activates PKC at the membrane, while IP_3_ diffuses and binds to IP_3_ receptors on the endoplasmic reticulum to mobilize stored calcium (Ca^2+^). These changes cause PIP_2_ depletion and increased intracellular concentration of Ca^2+^. This increased concentration of Ca^2+^ activates PKC‐dependent phosphorylation and forms a complex with http://www.guidetopharmacology.org/GRAC/LigandDisplayForward?ligandId=2351 (Ca^2+^CaM), both of which suppress http://www.guidetopharmacology.org/GRAC/FamilyIntroductionForward?familyId=81 (Figure 1), leading to depolarization and increased nociception (Brown & Passmore, 2009; Chen, Li, Hiett, & Obukhov, 2016).

#### 2.2.2 H_2_ receptor

**Figure 2:**
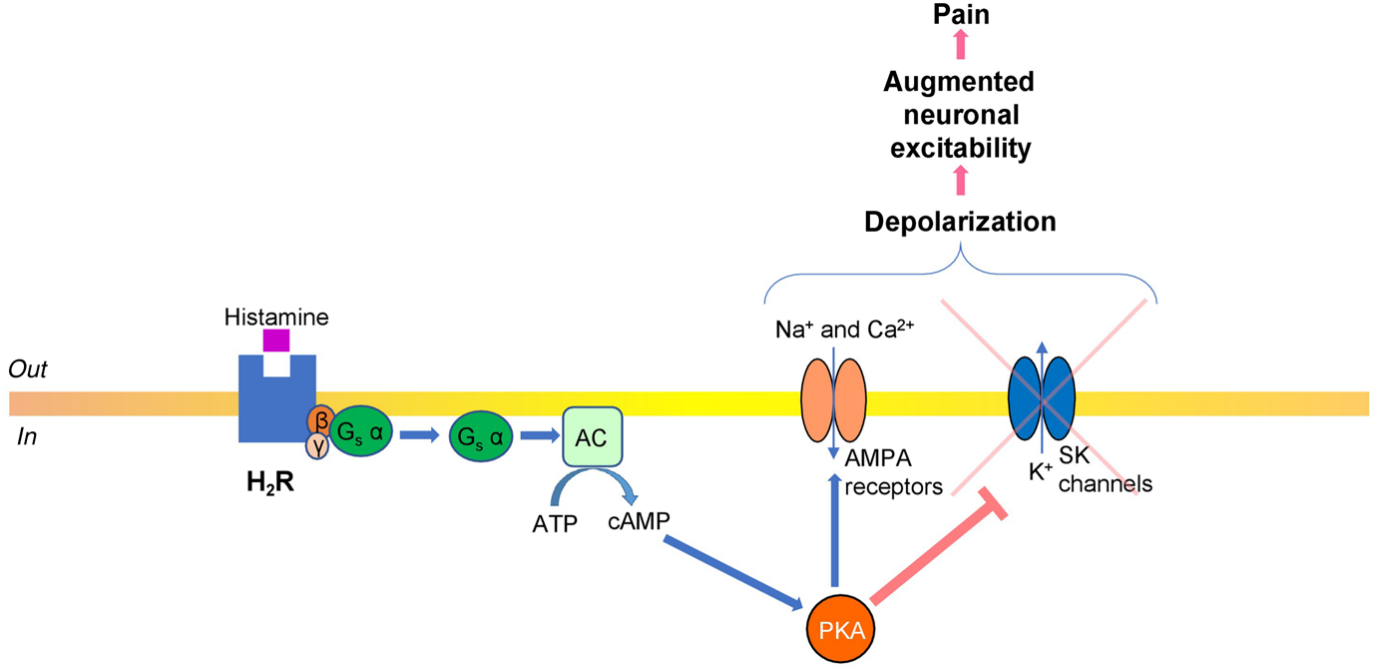
Diagram illustrating histamine receptor signalling—G_s_ pathway. Histamine binds to the H_2_ receptor subtype that is coupled with the G_s_‐type protein. G_s_ α subunit activates AC, with subsequent production of cAMP, which then stimulates PKA. PKA‐dependent phosphorylation activates ligand gated α‐AMPA receptors, which open and facilitate influx of Na^+^ and, less commonly, Ca^2+^. PKA also suppresses K^+^ efflux through small conductance Ca^2+^‐activated potassium channels (SK channels). Both the activation of AMPA receptors and inhibition of SK channels depolarize the neurons, with consequent augmentation of their excitability and increased pain signalling

H_2_ receptors are postsynaptic, predominantly associated with http://www.guidetopharmacology.org/GRAC/FamilyDisplayForward?familyId=257 via coupling to G_s_ and, in a similar fashion to H_1_ and H_3_ receptors, G_q_ proteins, both pathways initiating excitatory downstream signalling. Thus, H_2_ receptor inhibition yielded efficient antinociceptive effects (Mojtahedin, 2016). G_s_ α subunit stimulates AC with consequent augmented production of cAMP and consequent activation of http://www.guidetopharmacology.org/GRAC/FamilyDisplayForward?familyId=284 (Brown et al., 2001). Elevated cAMP concentration up‐regulates PKA, which as reported for hippocampal neurons, could activate ligand gated http://www.guidetopharmacology.org/GRAC/FamilyDisplayForward?familyId=75 with resultant depolarization and increase of neuronal firing (Park et al., 2016). Also, as it was reported for after‐hyperpolarization in enteric neurons, PKA inhibits small conductance http://www.guidetopharmacology.org/GRAC/FamilyDisplayForward?familyId=69 with resultant augmented neuronal excitability (Figure 2; Vogalis, Harvey, & Furness, 2003).

#### 2.2.3 H_3_ receptor

**Figure 3:**
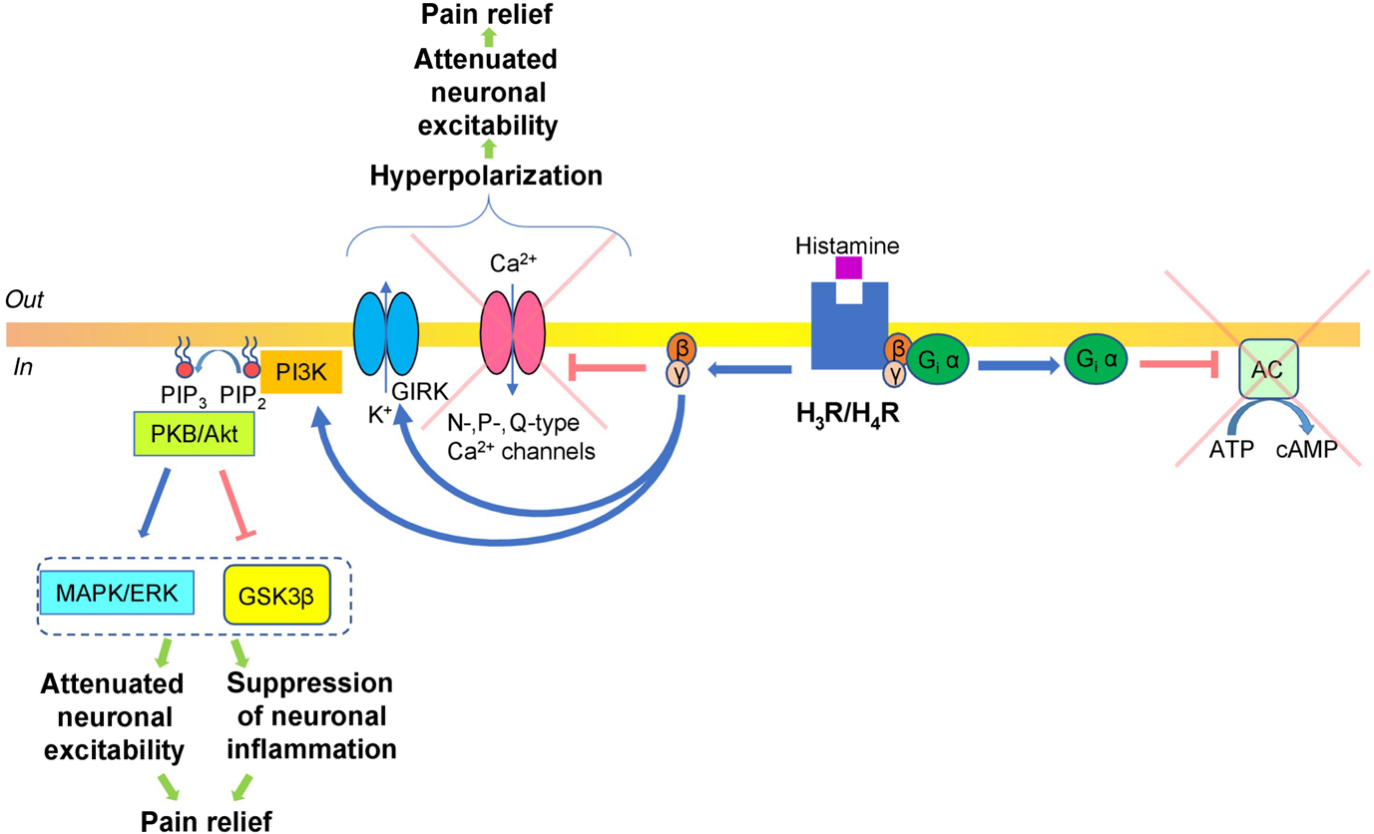
Diagram illustrating histamine receptor signalling—G_i_ pathway. Histamine binds to the histamine receptors (presynaptic and postsynaptic H_3_ or H_4_ receptor subtypes) that are coupled with G_i_‐type protein. The G_i_ α subunit inhibits AC with subsequent suppression of cAMP production and inhibition of PKA activity. Also, G_i_ β and γ subunits can inhibit Ca^2+^ influx through voltage‐gated N‐, P‐, and Q‐type Ca^2+^ channels and stimulate G protein‐coupled inwardly rectifying potassium (GIRK) channels, with resultant K^+^ efflux. Both effects on N‐type Ca^2+^ and GIRK channels result in the development of hyperpolarization, attenuation of neuronal excitability, and resultant pain relief. Besides G_i_ α subunit effects, H_3_ receptor activation could produce analgesic effects through G_i_ β and γ subunits, which up‐regulate the PI3K pathway, with the subsequent production of phosphatidylinositol 2,4,5‐trisphosphate (PIP_3_) from phosphatidylinositol 4,5‐bisphosphate (PIP_2_). PIP_3_ recruits PKB (Akt), which phosphorylates and inactivates glycogen synthase kinase 3β (GSK3β). In parallel, PKB (Akt)‐dependent phosphorylation additionally activates the MAPK/ERK cascade. The action on both GSK3β and MAPK/ERK decreases neuronal excitability, inhibits mechanisms of neuronal inflammation, and, therefore, produces pain relief

Presynaptic H_3_ receptors are coupled with G_i_ (AC inhibitory) proteins (Nieto‐Alamilla et al., 2016; Schlicker & Kathmann, 2017). G_i_ α subunit‐mediated AC inhibition results in a decreased intracellular concentration of cAMP and subsequent down‐regulation of PKA (Nieto‐Alamilla et al., 2016). In sympathetic and sensory neurons, it was reported that H_3_ receptor activation stimulated dissociation of G_i_ β and γ subunits from G_i_ α subunit, which then inhibited voltage‐gated Ca^2+^ influx through N‐, P‐, and Q‐type Ca^2+^ channels (Zamponi & Currie, 2013), and stimulated http://www.guidetopharmacology.org/GRAC/FamilyIntroductionForward?familyId=74 (Luscher & Slesinger, 2010). Both mechanisms could hyperpolarize presynaptic neurons, reduce neuronal excitability, and produce pain relief (Figure 3).

Besides the G_i_ pathway, postsynaptic activation of H_3_ receptors was reported to stimulate PLC in a subpopulation of striatal neurons, with subsequent activation of the IP_3_ pathway followed by increased intracellular concentrations of Ca^2+^ (Rivera‐Ramírez et al., 2016). Thus, it is analogous to the mechanisms described for H_1_ /H_2_ receptors coupled to G_q_ proteins (Figure 1). Similarly, the H_1_ and H_2_ receptor G_q_ cascade PLC signalling pathways modulate neuronal excitability with resultant potential facilitation of pain sensitivity. Furthermore, H_3_ receptor activation was established to inhibit http://www.guidetopharmacology.org/GRAC/ObjectDisplayForward?objectId=2030 (GSK3β) and MAPK/http://www.guidetopharmacology.org/GRAC/FamilyDisplayForward?familyId=514 pathways (Bongers et al., 2007; Morita, Aida, & Miyamoto, 1983). These effects are translated via G_i_ β and γ subunits (Lai et al., 2016), which up‐regulate the http://www.guidetopharmacology.org/GRAC/FamilyDisplayForward?familyId=781 pathway with subsequent production of http://www.guidetopharmacology.org/GRAC/LigandDisplayForward?ligandId=2353 (PIP_3_) from PIP_2_, which results in the recruitment of http://www.guidetopharmacology.org/GRAC/FamilyDisplayForward?familyId=285 (Akt). PKB is initially activated by http://www.guidetopharmacology.org/GRAC/ObjectDisplayForward?objectId=1519 (PDK1) and http://www.guidetopharmacology.org/GRAC/ObjectDisplayForward?objectId=2109 complex 2‐dependent phosphorylation (Dzamko, Zhou, Huang, & Halliday, 2014). PKB, via phosphorylation, inactivates GSK3β, which decreases neuronal excitability (Paul et al., 2016), inhibits neuronal inflammation (Maixner & Weng, 2013), and, subsequently, relieves pain. PKB‐dependent phosphorylation that activates the MAPK/ERK cascade was also reported to be a highly efficient neuroprotective mechanism for chronic inflammatory and neuropathic pain (Cruz & Cruz, 2007; Figure 3).

#### 2.2.4 H_4_ receptor

The role of H_4_ receptors in the nervous system is poorly understood (Schneider & Seifert, 2016). H_4_ receptors are known to be coupled to G_i_ proteins, and their downstream pathways are postulated to be similar to those described for H_3_ receptors (Figure 3). Compared to the other three types of histamine receptors, the H_4_ receptor is not expressed abundantly in the CNS and PNS. By quantitative single‐cell Ca^2+^ imaging, it was demonstrated that histamine induces a Ca^2+^ increase in a subset of sensory neurons (3–10%) via activation of the H_1_ and H_4_ receptors as well as inhibition of the H_3_ receptor. It is assumed that the decreased threshold in response to H_3_ receptor antagonism, which accounts for the analgesic effect of H_3_ receptor antagonists, activates H_1_ and H_4_ receptors on sensory neurons, which in turn results in the excitation of histamine‐sensitive afferents and, therefore, may result in a modulation of pain sensitivity (Rossbach et al., 2011).

## 3 HISTAMINE RECEPTOR LIGANDS AND NEUROPATHIC PAIN

### 3.1 H_1_ and H_2_ receptor ligands and neuropathic pain

The best‐known roles for the H_1_ receptor are regulation of vasodilation and bronchoconstriction on multiple cell types, including endothelial and smooth muscle cells, while the H_2_ receptor is primarily involved in the modulation of gastric secretion on parietal cells (Barocelli & Ballabeni, 2003; Simons, 2003). There is also evidence for their expression in the nervous system, where they regulate some neuronal functions (Haas et al., 2008; Kashiba, Fukui, & Senba, 2001; Murakami et al., 1999).

Both H_1_ and H_2_ receptors have been implicated in the role of histamine in nociception and chronic pain (Table 1). Interestingly, with the discovery of H_1_ and H_2_ receptor ligands in the 1950s, controlled clinical studies using these H_1_ and H_2_ receptor antagonists reported mild analgesic activity and their potential as analgesic adjuvants, particularly in conditions where pain was induced by histamine. Most of the clinical studies focused on http://www.guidetopharmacology.org/GRAC/LigandDisplayForward?ligandId=1224 (first generation H_1_ receptor antagonist) and showed its analgesic potential in the treatment of dysmenorrhea, atypical head and face pain, trigeminal neuralgia, and thalamic syndrome (Rumore & Schlichting, 1986). In addition, diphenhydramine, when combined with opioids, showed its potential as an analgesic adjuvant in refractory cancer pain (Santiago‐Palma, Fischberg, Kornick, Khjainova, & Gonzales, 2001). In addition to clinical evidence for the analgesic potential of H_1_ and H_2_ receptor antagonists, preclinical studies identified the expression of H_1_ and H_2_ receptors in nociceptive pathways and, therefore, further supported the roles of H_1_ and H_2_ receptors in the regulation of pain. There are limited anatomical data available for H_2_ receptors, despite the report of H_2_ receptor mRNA expression in human spinal cord (Murakami et al., 1999). The potential involvement of H_1_ receptors in the modulation of neuropathic pain has been investigated more extensively. In studies using in situ hybridization techniques in the guinea pig, the H_1_ receptor mRNA was shown to be expressed in about 15–20% of the central trigeminal and lumbar dorsal root ganglion (DRG) neurons. These sensory neurons are fundamental to nociceptive processes, potentially responding to histamine by acting on H_1_ receptors. These neurons are exclusively small in size and coexpress isolectin B4, but not http://www.guidetopharmacology.org/GRAC/LigandDisplayForward?ligandId=2098 (SP) or http://www.guidetopharmacology.org/GRAC/LigandDisplayForward?ligandId=3586, suggesting characteristics of unmyelinated C‐fibres involved in acute nociception. Interestingly, the potential role of H_1_ receptors in the regulation of neuropathic pain sensitivity can be explained by the marked (up to fourfold) increase in H_1_ receptor expression in the mainly small‐sized DRG neurons, 1–5 days after a crush injury of the sciatic nerve. Moreover, this study showed new characteristics of peptidergic (SP/CGRP) sensory neurons not detected prior to nerve injury, suggesting that the sensory modalities evoked by histamine acting via H_1_ receptors in normal and neuropathic pain states may result in different effects. This demonstration of the potential up‐regulation of H_1_ receptor number in injured afferent nerves further supports the involvement of H_1_ receptors in the regulation of neuropathic pain hypersensitivity, presumably expressed on unmyelinated C‐fibres (Kashiba et al., 2001). The earliest electrophysiological study to probe the histaminergic system in neuropathic pain transmission reported that daily injections of http://www.guidetopharmacology.org/GRAC/LigandDisplayForward?ligandId=2603, a CNS‐sparing H_1_ receptor antagonist, acting via peripheral histamine‐sensitive C‐fibres, blocked ectopic spontaneous discharges from the neuroma and suppressed autotomy following sciatic and saphenous neurectomy (Seltzer et al., 1991; Yu et al., 2013). More recently, i.c.v. injection of histamine blocked mechanical and thermal hypersensitivity associated with neuropathic pain (Sanna et al., 2015; Wei et al., 2016). However, these pain‐modulatory effects of histamine can vary depending on the dose of histamine administered i.c.v., due to an action on H_2_ receptors and the involvement of adrenoceptors (Wei et al., 2014; Wei et al., 2016).

**Table 1:** A summary of the effects produced by histamine receptor ligands in animal models of neuropathic pain

Drug	Model	Strain	Test	Effect	Reference
H_1_ antagonists					
Astemizole	Neurectomy	Sabra rats	Score	i.p. suppressed autotomy	Seltzer, Paran, Eisen, & Ginzburg, 1991
Chlorpheniramine	TNT	Wistar rats	Acetone	i.p. reduced allodynia and prevented cold plate avoidance behaviour	Khalilzadeh et al., 2018
			von Frey		
			Double plate		
			Open field		
	PLSN	Wistar rats	Hargreaves	i.p. suppressed and alleviated hyperalgesia	Zuo et al., 2003
			Randall‐Selito		
Fexofenadine	TNT	Wistar rats	Acetone	p.o. reduced allodynia and prevented cold plate avoidance behaviour	Khalilzadeh et al., 2018
			von Frey		
			Double plate		
			Open field		
http://www.guidetopharmacology.org/GRAC/LigandDisplayForward?ligandId=7216	Peripheral axotomy	Sprague–Dawley rats	Score of autotomy	i.p. did not block analgesic effects of histidine on pain behaviour but alone suppressed autotomy	Yu et al., 2013
Mepyramine (pyrilamine)	PLSN	Sprague–Dawley rats	von Frey	Intrathecally, i.c.v. blocked analgesic effects of histidine on pain behaviour	Yu et al., 2016
			IR laser		
	SNL	Hannover‐Wistar rats	von Frey	Intrathecally did not attenuate the antihypersensitivity effect of histamine	Wei, Viisanen, You, & Pertovaara, 2016
			Radiant heat		
	SNL	Hannover‐Wistar rats	von Frey test	Into LC did not attenuate the antihypersensitivity effect of histamine and alone failed to influence pain	Wei, Jin, Viisanen, You, & Pertovaara, 2014
			Radiant heat		
	PLSN	Rats	von Frey	i.c.v was ineffective	Huang, Adachi, Nagaro, Liu, & Arai, 2007
http://www.guidetopharmacology.org/GRAC/LigandDisplayForward?ligandId=7282	Vincristine induced	Wistar albino rats	Pinprick	i.p. reduced hyperalgesia and allodynia	Jaggi, Kaur, Bali, & Singh, 2017
			Acetone		
			Hot plate		
H_2_R antagonists					
Cimetidine	PLSN	Sprague–Dawley rats	von Frey	Intrathecally, i.c.v. did not block histidine's analgesic effects on pain behaviour	Yu et al., 2016
			IR laser		
	PLSN	Wistar rats	Hargreaves	i.p. suppressed and alleviated hyperalgesia	Zuo et al., 2003
			Randall‐Selito		
Famotidine	TNT	Wistar rats	Acetone	p.o. was ineffective in all tests	Khalilzadeh et al., 2018
			von Frey		
			Double plate		
			Open field		
	PLSN	Sprague–Dawley rats	von Frey	i.p. reduced allodynia and hyperalgesia	Yue et al., 2014
			Diode laser		
Ranitidine	TNT	Wistar rats	Acetone	i.p. reduced allodynia and prevented cold plate avoidance behaviour	Khalilzadeh et al., 2018
			von Frey		
			Double plate		
			Open field		
	Vincristine induced	Wistar rats	Pinprick	i.p. reduced hyperalgesia and allodynia	Jaggi et al., 2017
			Acetone		
			Hot plate		
	PLSN	Rats	von Frey	i.c.v. increased hypersensitivity	Huang et al., 2007
Zolantidine	SNL	Hannover‐Wistar rats	von Frey	i.t. attenuated the antihypersensitivity effect of histamine	Wei et al., 2016
			Radiant heat		
	SNL	Hannover‐Wistar rats	von Frey	Into LC attenuated histamine's analgesic effect but alone failed to influence pain	Wei et al., 2014
			Radiant heat		
H_3_R antagonists and H_3_R antagonists/inverse agonist					
A‐960656	SNL	Hannover‐Wistar rats	von Frey	Into LC reduced hypersensitivity	Wei et al., 2014
			Radiant heat		
	SNL	Hannover‐Wistar rats	von Frey	i.t. reduced hypersensitivity	Wei et al., 2016
			Radiant heat		
	SNL	Sprague–Dawley rats	von Frey	p.o. reduced hypersensitivity	Cowart et al., 2012
http://www.guidetopharmacology.org/GRAC/LigandDisplayForward?ligandId=1265	SNI	BL6 mice	Hargreaves	i.p. was ineffective	Zhang et al., 2012
			Dynamic plantar aesthesiometer		
E‐162	CCI	Swiss CD1 mice	von Frey	i.p. reduced hypersensitivity	Popiolek‐Barczyk et al., 2018
			Cold plate		
			Tail‐flick		
GSK189254	CCI	Random‐hooded rats	von Frey	p.o. reduced allodynia and hyperalgesia	Medhurst et al., 2008
			Randall‐Selito		
	SNL	Sprague–Dawley rats	von Frey	i.p. reduced allodynia	Hsieh et al., 2010
GSK334429	CCI	Random‐hooded rats	von Frey	p.o. reduced allodynia and hyperalgesia	Medhurst et al., 2008
			Randall‐Selito		
Pitolisant	SNI	BL6 mice	Hargreaves	i.p. was ineffective, high doses increased thermal but not mechanical hypersensitivity	Zhang et al., 2012
			Dynamic plantar aesthesiometer		
S38093	CCI and oxaliplatin induced	Sprague–Dawley rats	Randall‐Selito	p.o. reduced hypersensitivity	Chaumette et al., 2018
			Tail‐immersion (10°C)		
ST‐889	SNI	BL6 mice	Hargreaves	i.p. was ineffective	Zhang et al., 2012
			Dynamic plantar aesthesiometer		
Thioperamide	PLSN	Rats	von Frey	i.c.v. increased but i.p. reduced hypersensitivity	Huang et al., 2007
	PLSN	Sprague–Dawley rats	Randall‐Selito	i.p. increased hypersensitivity	Smith et al., 2007
H_4_R agonists					
ST‐1006	SNI	CD1 mice	Hargreaves	i.c.v. reduced allodynia and hyperalgesia	Sanna et al., 2015
			Dynamic plantar aesthesiometer		
VUF‐8430	SNI	CD1 mice	Hargreaves	i.c.v. reduced allodynia and hyperalgesia	Sanna et al., 2015
			Dynamic plantar aesthesiometer		
	SNI	CD1 mice	Hargreaves	i.t.reduced allodynia and hyperalgesia	Sanna, Lucarini, et al., 2017
			Dynamic plantar aesthesiometer		
	PLSN	Sprague–Dawley rats	Randall‐Selito	i.p. reduced hypersensitivity	Smith et al., 2007
H_4_R antagonists					
JNJ7777120	PLSN	Sprague–Dawley rats	Randall‐Selito	i.p. increased hypersensitivity	Smith et al., 2007
	SNL	Sprague–Dawley rats	von Frey	i.p. reduced allodynia	Hsieh et al., 2010
JNJ10191584	SNI	CD1 mice	Hargreaves	p.o. blocked the analgesic effect of i.c.v. ST‐1006 and VUF‐8430 and was ineffective alone	Sanna et al., 2015
			Dynamic plantar aesthesiometer		
	SNI	CD1 mice	Hargreaves	p.o. blocked the analgesic effect of i.t. VUF‐8430 and was ineffective alone	Sanna et al., 2015
			Dynamic plantar aesthesiometer		
TR‐7	CCI	Swiss CD1 mice	von Frey	i.p. reduced hypersensitivity	Popiolek‐Barczyk et al., 2018
			Cold plate		
			Tail‐flick		

Abbreviations: CCI, chronic constriction nerve injury; LC, locus coeruleus; PLSN, partial ligation of sciatic nerve; SNI, spared nerve injury; SNL, spinal nerve ligation; TNT, tibial nerve transection.

To further support the role of H_1_ and H_2_ receptors in the regulation of pain, separate studies using knockout (KO) mice lacking H_1_ and H_2_ receptors demonstrated that these mice displayed significantly lower responses to nociceptive stimuli when compared to their wild‐type controls (Mobarakeh et al., 2002; Mobarakeh, Takahashi, Sakurada, Kuramasu, & Yanai, 2006). Interestingly, the antinociceptive phenotype of H_2_ receptor KO mice was relatively less prominent when compared to H_1_ receptor KO mice, suggesting a potentially distinct role for these receptors in the modulation of pain. Indeed, behavioural studies using a model of neuropathic pain, induced by the partial ligation of the sciatic nerve, showed that the CNS‐permeable H_1_ receptor antagonist http://www.guidetopharmacology.org/GRAC/LigandDisplayForward?ligandId=1227, but not the H_2_ receptor CNS‐sparing antagonist http://www.guidetopharmacology.org/GRAC/LigandDisplayForward?ligandId=1231, blocked the effects of histidine on neuropathic pain hypersensitivity and spinal microglia activity (Yu et al., 2016). In addition, Jaggi et al. (2017) suggested that the H_1_ receptor plays a more important role in a vincristine‐induced model of neuropathic pain, when compared to H_2_ receptors. However, Khalilzadeh et al. (2018) observed different behavioural effects upon tibial nerve transection‐induced neuropathic pain with respect to the extent of brain penetration of the ligands, in a study focused on centrally active and centrally sparing H_1_ and H_2_ receptor antagonists. Specifically, both http://www.guidetopharmacology.org/GRAC/LigandDisplayForward?ligandId=6976, a centrally and peripherally active H_1_ receptor antagonist, and http://www.guidetopharmacology.org/GRAC/LigandDisplayForward?ligandId=4819, an H_1_ receptor, centrally sparing antagonist, were found to profoundly decrease the mechanical hypersensitivity associated with the development of neuropathic pain. In contrast, while http://www.guidetopharmacology.org/GRAC/LigandDisplayForward?ligandId=1234, a widely used centrally permeable H_2_ receptor antagonist, also improved mechanical hypersensitivity, http://www.guidetopharmacology.org/GRAC/LigandDisplayForward?ligandId=7074, a centrally sparing H_2_ receptor antagonist, was ineffective. These results indicate that both blood brain barrier penetrating and poorly penetrating histamine H_1_ receptor antagonists can block neuropathic pain hypersensitivity, but only the blood brain barrier penetrating histamine H_2_ receptor antagonist can generate an analgesic effect in neuropathic pain. In line with this observation, histamine‐induced mechanical hypersensitivity was prevented by spinal pretreatment with zolantidine, a brain penetrating H_2_ receptor antagonist, as well as localized peripheral administration of cimetidine (H_2_ receptor antagonist) and chlorpheniramine (H_1_ receptor antagonist) into the plantar side of the hindpaw (Zuo et al., 2003).

Taken together, these results indicate that the brain histamine, acting particularly via central H_1_ and H_2_ receptors, may be involved in the modulation of neuropathic pain. These studies consistently support the idea that CNS‐permeable H_1_ and H_2_ receptor antagonists may potentially be used as analgesics for patients with neuropathic pain. The involvement of central H_2_ receptors in the regulation of neuropathic pain hypersensitivity was also demonstrated in studies where histamine (presumably postsynaptically‐ induced) facilitated mechanical hypersensitivity mediated by http://www.guidetopharmacology.org/GRAC/FamilyDisplayForward?familyId=75 receptors as well as, in a dose‐dependent manner, http://www.guidetopharmacology.org/GRAC/ObjectDisplayForward?objectId=585 channel expression in primary afferent neurons in the sciatic nerve and L4/L5 DRG (Wei et al., 2016; Yue et al., 2014). While sodium channels are responsible for the development and maintenance of neuropathic pain (Ossipov & Porreca, 2005; Yue et al., 2014), the above studies highlight the importance of histamine acting via H_2_ receptors in the regulation of mechanisms associated with neuropathic pain states. The influence of the H_2_ receptor on non‐neuronal cells (mast cells) is discussed later in this review.

### 3.2 H_3_ receptor ligands and neuropathic pain

H_3_ receptors are mostly presynaptic, expressed as autoreceptors on histaminergic neurons involved in the negative feedback control of histamine levels (Arrang et al., 1983; Hough & Rice, 2011), while H_3_ heteroreceptors on postsynaptic nonhistaminergic neurons also regulate negatively the release of neurotransmitters, such as http://www.guidetopharmacology.org/GRAC/LigandDisplayForward?ligandId=294, http://www.guidetopharmacology.org/GRAC/LigandDisplayForward?ligandId=940, 5‐HT, and http://www.guidetopharmacology.org/GRAC/LigandDisplayForward?ligandId=484 (Blandina, Munari, Giannoni, Mariottini, & Passani, 2010; Gemkow et al., 2009; Giannoni et al., 2010). Since the cloning of H_3_ receptors (Lovenberg et al., 1999), there has been an increased interest within the pharmaceutical industry in developing ligands for this receptor to target several diseases, including neuropathic pain. This interest was strongly fuelled by the report of H_3_ receptor expression in nociceptive pathways, suggesting its functional involvement in the regulation of nociceptive transmission (Cannon et al., 2007). Indeed, the histamine H_3_ receptor consists of several functional isoforms expressed in both the CNS and PNS, particularly along the ascending nociceptive pathway and descending pain‐control pathway, that are critical for the processing of nociceptive information. Within the CNS, this receptor has been found in various brain areas, such as thalamus, hypothalamus, prefrontal cortex, and periaqueductal grey area (Drutel et al., 2001), and in the spinal cord (Cannon et al., 2007; Heron, Rouleau, Cochois, Pillot, & Schwartz, 2001; Medhurst et al., 2008). In the periphery, the expression of H_3_ receptors has been identified in DRG, superior cervical ganglia, and dermal tissues (Cannon et al., 2007; Medhurst et al., 2008).

However, while the localization of H_3_ receptors strongly suggests its functional involvement in the regulation of nociceptive transmission, pharmacological studies using agonists and antagonists of H_3_ receptors are confusing as these drugs have different effects on the nociceptive threshold depending on the pain model used, the nociceptive stimulus selected, together with the affinity and selectivity for the histamine receptors, and the dose and routes of administration (Huang et al., 2007; Smith et al., 2007). Several studies have reported inhibitory effects on pain following activation of H_3_ receptors using agonists (Cannon et al., 2003; Hasanein, 2011). The involvement of H_3_ receptors in neuropathic pain has been implicated using a range of H_3_ receptor antagonists/inverse agonists (Table 1). The antagonism at H_3_ receptors results in reduced mechanical and cold hypersensitivity associated with neuropathic pain (chronic constriction injury model or spinal nerve ligation model) as shown in studies using E‐162 or GSK189254, selective H_3_ receptor antagonists, where its strong analgesic effect was observed after a single systemic (i.p.) dose (Hsieh et al., 2010; Popiolek‐Barczyk et al., 2018). In addition, repeated, orally delivered doses of GSK189254, http://www.guidetopharmacology.org/GRAC/LigandDisplayForward?ligandId=9103, S38093, and A‐960656, selective H_3_ receptor antagonists/inverse agonists, significantly reduced paw withdrawal threshold to mechanical stimuli or elicited an analgesic effect in the vocalization test of neuropathic pain (chronic constriction injury model or spinal nerve ligation model), showing comparable efficacy to http://www.guidetopharmacology.org/GRAC/LigandDisplayForward?ligandId=5484 or http://www.guidetopharmacology.org/GRAC/LigandDisplayForward?ligandId=5483, which are both used clinically as first‐line treatments (Chaumette et al., 2018; Cowart et al., 2012; Medhurst et al., 2008). The analgesic efficacy of S38093 was also confirmed in other models of neuropathic pain with different aetiologies, such as diabetic and chemotherapeutic agent‐induced neuropathy, where the drug again showed analgesic potency similar to pregabalin and gabapentin (Chaumette et al., 2018). Analgesia induced by the blockade of the H_3_ receptor is possibly the result of the regulation of histamine levels in the CNS, as depolarization activates histamine synthesis in nerve endings, a process that is controlled by H_3_ autoreceptors (Arrang et al., 1983; Hough & Rice, 2011). Indeed, Wei et al. (2016) proposed that blocking the autoinhibitory H_3_ receptor on histaminergic terminals in the pontine locus coeruleus (LC), which receives efferent projections from the histaminergic tuberomammillary nucleus, facilitated endogenous release of histamine leading to neuropathic hypersensitivity inhibition through the regulation of descending noradrenergic pathways. In addition, there is accumulating evidence to support the idea that the analgesic effects of H_3_ receptor antagonists/inverse agonists in neuropathic pain can be partially mediated by http://www.guidetopharmacology.org/GRAC/FamilyDisplayForward?familyId=4 desensitization (induced by H_3_ receptor antagonists/inverse agonists) in the LC and spinal cord. This suggests an inhibitory role for the central heteroreceptor noradrenergic transmission in the efficacy of H_3_ receptor antagonists/inverse agonists. In agreement with this idea, systemic administration of α_2_ adrenoceptor agonists or nerve injury‐induced activation of these α_2_ adrenoceptors decreases the firing activity of LC noradrenergic cells, resulting in the dampening of noradrenaline release in the terminal area (e.g., prefrontal cortex or spinal cord) and promoting neuropathic hypersensitivity by attenuating descending inhibition (Chaumette et al., 2018; Wei et al., 2010; Wei et al., 2014). In contrast, treatment with H_3_ receptor antagonists/inverse agonists restores LC and decreases α_2_ adrenoceptor activity, respectively, potentially leading to relief in neuropathic pain hypersensitivity (Chaumette et al., 2018; Wei et al., 2010). To further support this proposed mechanism, it was shown that bilateral lesion of the LC, transection of the spinal cord, or direct injection of a α_2_ agonist (fadolmidine) into the LC reversed the antihyperalgesic effect produced by H_3_ receptor antagonists, A‐960656 or GSK189254 (Chaumette et al., 2018; McGaraughty, Chu, Cowart, & Brioni, 2012; Wei et al., 2014). In line with this, electrophysiological studies performed in anaesthetized animals indicated that, after systemic administration, GSK189254 dose‐dependently decreased both evoked and spontaneous firing of wide dynamic range neurons in neuropathic, but not sham‐operated rats (McGaraughty et al., 2012). However, analgesia induced by the blockade of the H_3_ receptor can also be mediated via H_3_ heteroreceptors that regulate other neurotransmitters' release; the blockade of the H_3_ receptor is known to increase the release of ACh, dopamine, 5‐HT, noradrenaline, and SP in the CNS (Blandina et al., 2010; Gemkow et al., 2009; Giannoni et al., 2010).

Interestingly, the majority of the behavioural observations published indicate that H_3_ receptor antagonists/inverse agonists do not produce any antinociceptive effects in *naïve* rodents, suggesting a possibility that H_3_ receptors are not involved or tonically activated in nociception (at least in relation to acute mechanical nociception), but are critical for pathological pain states, particularly for mechanical hypersensitivity (Chaumette et al., 2018; McGaraughty et al., 2012). Also, H_3_ receptor KO mice showed unaltered response to mechanical pinch (Cannon et al., 2003), and multiple studies suggest modality (mechanical vs. heat) and intensity (preferential responses to low‐intensity tail pinch stimulation; Cannon et al., 2003) with specific antinociceptive effects mediated by H_3_ receptors. H_3_ receptor antagonists/inverse agonists at a dose that produced a significant reduction of mechanical hypersensitivity in neuropathic pain did not attenuate heat hypersensitivity indicating that the antihyperalgesic effect was due to selective depression of spinal sensory rather than motor neurons (Wei et al., 2014; Wei et al., 2016). To support this, in situ hybridization studies revealed H_3_ receptor mRNA transcripts in the sensory neurons of the dorsal horn and DRG (Heron et al., 2001). Moreover, receptor autoradiography studies, using [^3^H] GSK189254, showed specific H_3_ receptor binding sites in the dorsal horn of the spinal cord and DRG, confirming these as sites of action of H_3_ receptor antagonists within structures receiving histaminergic innervation and are critical for processing of pain information (Medhurst et al., 2008). The modality‐ and intensity‐specific antinociceptive effects of H_3_ receptor activation/inhibition may also suggest involvement of a specific population of sensory fibres that regulate mechanical hypersensitivity. In line with this, immunohistochemical studies identified localization of H_3_ receptors (confirmed by H_3_ receptor KO mice) on medium‐size cell bodies in DRG and on small‐calibre periarterial, peptidergic Aδ fibres that ramified in dorsal horn laminae I, II, and V and coexpress immunoreactivity for acid‐sensing ion channel 3 and 200‐kD neurofilament protein. This strongly supports the involvement of H_3_ receptors in the regulation of mechanical sensitivity (Cannon et al., 2007). In addition, Medhurst et al. (2007) showed that GSK207040 and GSK334429, selective H_3_ receptor antagonists, blocked the secondary mechanical allodynia in the capsaicin‐induced model of pain. Secondary mechanical hypersensitivity is known to be exclusively signalled by A‐fibres and amplified by sensitized dorsal horn neurons (Magerl, Fuchs, Meyer, & Treede, 2001; Treede & Magerl, 2000). Thus, presumably reducing the sensitivity of H_3_ receptor‐positive A‐fibres with selective H_3_ receptor antagonists resulted in a diminished input to the dorsal horn and the subsequent amplification of the A‐fibres response, confirming the potential role for H_3_ receptors in the modulation of central sensitization processes. In contrast, thermal (heat) hypersensitivity is generally regarded as a sign of the peripheral sensitization of C‐fibres, which do not express H_3_ receptors (Cannon et al., 2007; Gold & Gebhart, 2010). The only study that reported a significant increase in thermal (radiant heat in Hargreaves test), but not mechanical, threshold in the spared nerve injury model of neuropathic pain used the selective H_3_ receptor inverse agonist pitolisant (Wakix™). The drug produced this unexpected effect at a dose 5× higher than its clinically relevant dose, and pharmacological analysis of this effect suggested at least partial involvement of transient receptor potential cation channel subfamily V member 1 (http://www.guidetopharmacology.org/GRAC/ObjectDisplayForward?objectId=507), without any contribution of H_3_ receptors (Zhang et al., 2012). Interestingly, the H_3_ receptor antagonist/inverse agonist E‐162, at a dose that produced a significant reduction in mechanical hypersensitivity, also attenuated the response to cold in neuropathic pain (Popiolek‐Barczyk et al., 2018). The signalling of cool temperatures that become aversive in neuropathic pain is known to be mediated via the http://www.guidetopharmacology.org/GRAC/ObjectDisplayForward?objectId=500 receptor, a member of the TRP channel family (Knowlton et al., 2013). It was reported that the number of TRPM8‐positive Aδ‐fibres (but not C‐fibres) increases after nerve injury (Ji, Zhou, Kochukov, Westlund, & Carlton, 2007); thus, it is possible that H_3_ receptor‐positive A‐fibres are probably sensitive to cooling and may contribute to cold hypersensitivity in neuropathic pain.

The most significant inconsistencies in behavioural outcomes in neuropathic pain can be found in studies on the role of a first‐generation imidazole‐based molecule, http://www.guidetopharmacology.org/GRAC/LigandDisplayForward?ligandId=1267 (H_3_ receptor antagonist, H_3_ /H_4_ receptor inverse agonist), in the regulation of mechanical hypersensitivity in neuropathic pain. On the one hand, blocking H_3_ receptors (and H_4_ receptors) by thioperamide resulted in a significant enhancement of mechanical hyperalgesia in a rat model of neuropathic pain induced by partial ligation of the sciatic nerve. Specifically, i.c.v. (Huang et al., 2007) or s.c. injection (Smith et al., 2007) of thioperamide directly into the operated hindpaw resulted in a significantly reduced mechanical withdrawal threshold as compared to controls. On the other hand, systemic (i.p.) injection of thioperamide significantly increased mechanical withdrawal threshold indicating an analgesic effect (Huang et al., 2007). The reason for this discrepancy may lie in the drug's dual affinity for both H_3_ and H_4_ receptors (e.g., the effect of thioperamide on neurotransmitter release in the anterior hypothalamic area of rats is nonreversible by an H_3_ receptor agonist, suggesting the involvement of H_4_ receptors; Yamamoto, Mochizuki, Okakura‐Mochizuki, Uno, & Yamatodani, 1997) and on the behavioural effects resulting from the route (localized vs. systemic) and dose of thioperamide administration. In addition, the involvement of other histaminergic mechanisms of action in the behavioural effects produced by thioperamide is suggested by the observation that thioperamide increases the density of intact mast cells in the injured nerve (Smith et al., 2007). While nerve injury causes a decrease in mast cell numbers as a consequence of degranulation (Zuo et al., 2003), thioperamide's action leads to an opposite effect that may represent a prevention of mast cell degranulation and stabilization or redistribution of mast cells in the injured nerve that theoretically would result in the inhibition of hyperalgesia, rather than its enhancement. This effect could be linked to the observation that histamine (acting through H_1_ and H_3_ receptors) inhibits the release of the pro‐inflammatory cytokine http://www.guidetopharmacology.org/GRAC/LigandDisplayForward?ligandId=5074 from alveolar macrophages (Sirois, Ménard, Moses, & Bissonnette, 2000), and antagonism of H_3_ receptors on macrophages resulted in an increase in TNF‐α and, subsequent, enhancement of mechanical hyperalgesia (Smith et al., 2007).

Taken together, the interpretation of the thioperamide data is complicated further since the drug has high affinity, not only for H_3_ and H_4_ receptors but also for http://www.guidetopharmacology.org/GRAC/FamilyDisplayForward?familyId=68 (Leurs et al., 1995). Studies with more selective H_3_ receptor antagonists/inverse agonists suggest that these ligands may be beneficial for the improvement of mechanical and cold hypersensitivity associated with neuropathic pain, particularly given their ability to modulate histamine levels, as well as several neurotransmitters, including ACh, histamine, noradrenaline, dopamine, and SP. However, due to the wide presynaptic and postsynaptic distribution of H_3_ receptors throughout the CNS and PNS, more research is certainly needed to clarify the involvement of peripheral, spinal, and brain H_3_ receptors in various neuropathic pain states, thus determining their full potential in neuropathic pain.

### 3.3 H_4_ receptor ligands and neuropathic pain

The H_4_ receptor, which has low homology with other histamine receptors, can be primarily found in bone marrow, intestinal tissue, spleen, thymus, and also in various immune cells, such as T cells, mast cells, neutrophils, and eosinophils, showing modulatory effects on these cells, including activation, migration, and production of cytokines and chemokines, suggesting its principal role in the regulation of immune/inflammatory mechanisms (Takeshita, Sakai, Bacon, & Gantner, 2003; Zhu et al., 2001). Interestingly, recent reports also indicate the presence of H_4_ receptors on peripheral sensory nerves, in the DRG, with more intense staining of small‐ and medium‐diameter cells, and in the spinal cord, especially laminae I and II (Sanna, Lucarini, et al., 2017; Strakhova et al., 2009). This neuronal localization supports H_4_ receptors involvement in the regulation of neuronal function related to the modulation of nociceptive transmission (Sanna, Ghelardini, Thurmond, Masini, & Galeotti, 2017; Sanna, Lucarini, et al., 2017).

The involvement of H_4_ receptors in both acute (Galeotti, Sanna, & Ghelardini, 2013) and persistent inflammatory pain (Hsieh et al., 2010) is relatively well documented, and recently, the role of H_4_ receptors in the modulation of neuropathic pain was identified in H_4_ receptor‐KO mice through the observation that these animals, when subjected to neuropathic pain, induced by spared nerve injury of sciatic nerve, showed enhanced hypersensitivity to mechanical and thermal stimuli compared to wild‐type controls (Sanna, Ghelardini, et al., 2017). Interestingly, H_4_ receptor deficiency does not support a role for H_4_ receptors in the physiological maintenance of pain threshold, as H_4_ receptor‐KO mice did not show any change in thermal or mechanical nociceptive thresholds, suggesting that the H_4_ receptor is specifically involved in the regulation of hypersensitivity associated with pathological chronic pain induced by nerve injury (Sanna, Ghelardini, et al., 2017). This observation in H_4_ receptor‐KO neuropathic mice is particularly important as H_4_ receptor mRNA expression in humans and rodents supports their involvement in the regulation of neuronal function, including regulation of neuropathic pain. The controversy around the generation of consistently specific H_4_ receptor antibodies highlights the need for cautious interpretation of some of the immunohistochemical outcomes (Beermann, Seifert, & Neumann, 2012; Gutzmer et al., 2012; Schneider & Seifert, 2016). In line with the observation from H_4_ receptor KO mice, blockade of H_4_ receptors by the specific H_4_ receptor antagonist JNJ7777120, injected s.c. directly into the operated hindpaw, resulted in a significant increase in mechanical hyperalgesia compared to controls (Smith et al., 2007). Subsequently, activation of H_4_ receptors by localized administration of potent and selective agonists, http://www.guidetopharmacology.org/GRAC/LigandDisplayForward?ligandId=8983 (i.c.v.) and http://www.guidetopharmacology.org/GRAC/LigandDisplayForward?ligandId=1274 (i.c.v., intrathecally, and s.c. directly into the operated hindpaw), resulted in a significantly reduced mechanical and thermal withdrawal threshold in mice subjected to neuropathic pain induced by spared nerve injury or partial nerve ligation of the sciatic nerve (Sanna et al., 2015; Sanna, Lucarini, et al., 2017; Smith et al., 2007). The analgesia produced by VUF8430 has been shown to be associated with a reduction in neuroinflammation and oxidative stress mediated by neuronal H_4_ receptors in the spinal cord and sciatic nerve (Sanna, Lucarini, et al., 2017), and the involvement of H_4_ receptors in the behavioural effects produced by ST‐1006 and VUF8430 was confirmed with http://www.guidetopharmacology.org/GRAC/LigandDisplayForward?ligandId=1277, H_4_ receptor antagonist also known as VUF6002, which fully prevented the analgesic effects produced by these H_4_ receptor agonists (Sanna et al., 2015).

Interestingly, similar to the H_3_ receptor, pharmacological studies using agonists and antagonists of H_4_ receptors demonstrate that these drugs can have different effects on the nociceptive threshold depending on the routes of administration and target cells (Popiolek‐Barczyk et al., 2018; Sanna et al., 2015). In contrast to the studies above that used H_3_ receptor agonists/antagonists after localized application, the antagonism of H_4_ receptors produced by systemic administration resulted in the alleviation of mechanical and cold hypersensitivity associated with neuropathic pain (chronic constriction injury model). Studies using TR‐7, a selective H_4_ receptor antagonist, elicited a strong analgesic effect after a single systemic (i.p.) dose, which was as effective as http://www.guidetopharmacology.org/GRAC/LigandDisplayForward?ligandId=1627, a gold standard in pain treatment (Popiolek‐Barczyk et al., 2018). In addition, JNJ7777120 reduced mechanical hypersensitivity after a systemic (i.p.) administration in neuropathic pain (chronic constriction injury model and spinal nerve ligation model; Hsieh et al., 2010). Given that H_4_ receptors are expressed on the immune cells, in addition to the well‐documented involvement of H_4_ receptors in the regulation of immune/inflammatory mechanisms (Takeshita et al., 2003; Zhu et al., 2001), it is possible that the antinociceptive action of H_4_ receptor antagonists, particularly after systemic administration, may result from a reduction in ongoing inflammatory processes at the site of nerve injury, since the analgesic effect produced by JNJ7777120 was weaker (secondary) than its anti‐inflammatory effect (Hsieh et al., 2010). An underlying mechanism may be associated with stabilization of mast cells that are known to regulate the recruitment of neutrophils and macrophages and, subsequently, to modulate the development of hyperalgesia in neuropathic pain (Smith et al., 2007; Zuo et al., 2003). It should be further noted that similar observations have been described for the closely related H_3_ receptor.

The H_4_ receptor is known to activate the MAPK signalling pathway in mast cells (Desai & Thurmond, 2011). Interestingly, Sanna et al. (2015, 2018) also identified the effect of H_4_ receptor stimulation on the activity of the MAPK signalling pathway in neurons. They demonstrated that modulation of this signalling pathway within the neurons of the DRG, spinal cord, and sciatic nerve underpinned H_4_ receptor agonist‐induced antiallodynic activity. They also revealed that neuropathic pain hypersensitivity observed in H_4_ receptor‐KO mice is associated with an overactivation of the spinal ERK–http://www.guidetopharmacology.org/GRAC/ObjectDisplayForward?objectId=2734 pathway in DβH immunoreactive neurons, supporting a potential association between the noradrenergic system and H_4_ receptor‐mediated analgesia. In summary, increasing evidence arising from H_4_ receptor KO mice and the use of selective ligands support H_4_ receptor as an interesting neuronal target for the treatment of chronic, particularly neuropathic, pain.

## 4 HISTAMINE AND NON‐NEURONAL CELLS IN NEUROPATHIC PAIN

Following peripheral nerve injury, the immune system seems to play a vital role in the development of persistent inflammation and chronic neuropathic pain (Marchand, Perretti, & McMahon, 2005). Non‐neuronal astrocytes, satellite glia cells, microglia, and mast cells play key roles in communication between the immune system and the CNS via the production of neuroinflammatory mediators, including histamine, 5‐HT, chemokines, and growth factors (Zhuang, Gerner, Woolf, & Ji, 2005). The neuroimmune interactions between these two systems may reflect distinct roles in the development of chronic neuropathic pain (Zhao et al., 2017). Stimulation of H_1_ receptors via a PKC/MAPK/http://www.guidetopharmacology.org/GRAC/ObjectDisplayForward?objectId=2062 signalling pathway has recently been shown to elicit release of the key pro‐inflammatory cytokines http://www.guidetopharmacology.org/GRAC/LigandDisplayForward?ligandId=4974 and http://www.guidetopharmacology.org/GRAC/LigandDisplayForward?ligandId=4998 with, subsequent, regulation of http://www.guidetopharmacology.org/GRAC/LigandDisplayForward?ligandId=5026 release from astrocytes (reviewed recently in Jurič, Kržan, & Lipnik‐Stangelj, 2016). Satellite glial cells, prominent in the PNS, including the DRGs, with active roles in persistent neuropathic pain are also known to secrete the cytokine IL‐6 in the chronic constriction injury neuropathic pain model, but the identity profile of the histamine receptor in these cells has yet to be established (Dubový, Klusáková, Svíženská, & Brázda, 2010). Activated microglia also release a myriad of pro‐inflammatory cytokines, including notably, IL‐6, IL‐1β, and TNF‐α (Kempuraj et al., 2016; Mika, Zychowska, Popiolek‐Barczyk, Rojewska, & Przewlocka, 2013). H_3_ and H_4_ receptor activation of primary and clonal microglia has been shown to inhibit these cytokines (Ferreira et al., 2012).

Mast cells are professional cellular suppliers of histamine and contribute to the histamine‐based effects in neuropathic pain. For example, mast cell depletion prevented mechanical allodynia in a mouse model of postoperative pain (Kaur, Singh, & Jaggi, 2017). Recently, it was shown that the administration of http://www.guidetopharmacology.org/GRAC/LigandDisplayForward?ligandId=7121, a second‐generation H_1_ receptor antagonist and mast cell stabilizer, blocked the development of mechanical allodynia and inhibited mast cell degranulation in mice with http://www.guidetopharmacology.org/GRAC/LigandDisplayForward?ligandId=7433‐induced mechanical allodynia pain. The H_1_ and H_4_ receptors are likely molecular players in this process (Sakamoto, Andoh, & Kuraishi, 2016). For example, genetically silencing the H_4_ receptor inhibited the production of IL‐1β for human mast cells (Ebenezer, Prasad, Rajan, Thangam, & Transduction, 2018), and H_4_ receptor activation was shown to stimulate a number of cytokines, including IL‐6 (Jemima, Prema, & Thangam, 2014). In addition, while H_1_ and H_2_ receptor antagonism reduced hypersensitivity following nerve injury, it is possible that histamine released by mast cells contributes to the recruitment of neutrophils and macrophages in neuropathic pain and, acting via these histamine receptors, contributes to the regulation of hypersensitivity in this type of chronic pain (Jaggi et al., 2017; Smith et al., 2007; Zuo et al., 2003). There is an important aspect associated with H_2_ receptor antagonism, which should be considered for its therapeutic potential in neuropathic pain control. In vitro studies using CHO and HEK‐293 cells identified time‐ and dose‐dependent up‐regulation of H_2_ receptors upon long‐term exposure to H_2_ receptor antagonists (e.g., ranitidine), which may underlie the development of tolerance after prolonged clinical use of these ligands and result in the rebound hypersecretion of gastric acid and anaphylaxis that can occur after withdrawal of treatment (Allen, Chazot, & Dixon, 2018; Smit et al., 1996). Thus, side effects linked to pharmacological tolerance may potentially compromise long‐term efficacy and tolerability of H_2_ receptor antagonists in neuropathic pain. Little is known about the role of the H_3_ receptors in non‐neuronal cells in neuropathic pain states.

Overall, non‐neuronal cells play a key, but poorly, defined role in the mechanisms underlying histamine‐mediated neuropathic pain. We propose that neuronal H_1_ and H_4_ receptors (Ferreira et al., 2012) may orchestrate these mechanisms, with IL‐6 and IL‐1β cytokines as common denominator mediators. Further complications arise from the recent observation that activated mast cells trigger microglial activation (Zhang, Wang, Dong, Xu, & Zhang, 2016). These cell types and their interactions may potentially go some way to explain the paradoxical effects of histamine ligands, particularly for the H_4_ receptor, seen in animal pain models.

## 5 HISTAMINE AND ITS INTERACTION WITH OPIOID SYSTEM IN NEUROPATHIC PAIN

Interestingly, in neuropathic pain, high doses of opioids are required to achieve pain relief, and pharmacological tolerance to analgesic effect of opioids develops rapidly (Osikowicz, Mika, Makuch, & Przewlocka, 2008). This phenomenon significantly restricts the clinical usefulness of opioids. In addition, the misuse of and addiction to opioids, including prescription pain relievers, morphine and heroin, as well as synthetic opioids, such as fentanyl, is a serious international crisis that affects public health as well as social and economic welfare (Lipman & Webster, 2015).

An interaction between histaminergic and opioidergic systems within the CNS was suggested nearly 30 years ago, through an observation that morphine administration resulted in the release of histamine and its increased turnover in the periaqueductal grey (Nishibori, Oishi, Itoh, & Saeki, 1985), suggesting that analgesia produced by opioids may be associated with the stimulation of histamine receptors at the supraspinal level. There are also data suggesting that ligands of histamine receptors may modulate the analgesic action of opioids; however, the site and mode of this interaction differ between the spinal or supraspinal level, and depend on the subtype of histamine receptor involved (Mobarakeh et al., 2002; Mobarakeh et al., 2006; Mobarakeh, Takahashi, & Yanai, 2009). Specifically, a series of studies over the last two decades has shown that in H_1_, H_2_, or H_3_ receptor‐KO mice, morphine‐induced antinociception was significantly augmented when compared to the wild‐type controls in models of acute pain. H_1_ receptor‐KO mice showed a reduced spontaneous nociceptive threshold as they responded to significantly lower pain stimuli when compared to their controls (Mobarakeh et al., 2002), while thresholds for pain perception in H_2_ receptor‐KO mice were higher when compared to their corresponding controls (Mobarakeh et al., 2006). Intrathecal administration of morphine in H_1_ and H_3_ receptor‐KO mice and i.c.v. morphine injection in H_2_ receptor‐KO mice produce enhanced analgesic effects (Mobarakeh et al., 2002; Mobarakeh et al., 2006). Interestingly, pharmacological blockade of H_1_ and H_3_ receptors by either intrathecal administration of the first‐generation antihistamine chlorpheniramine (H_1_ receptor antagonist) or thioperamide (H_3_ receptor antagonist, H_3_ /H_4_ receptor inverse agonist), or H_2_ receptor antagonism, produced by zolantidine (i.c.v. route), resulted in the potentiation of the morphine analgesic effect (Mobarakeh et al., 2002; Mobarakeh et al., 2006). These behavioural studies, in both KO mice and involving pharmacological interventions, clearly demonstrated that blocking H_1_, H_2_ and H_3_ receptors in combination with morphine may have beneficial effects on analgesia and suggested that endogenous histamine may exert an inhibitory effect on morphine‐induced analgesia acting via H_1_ and H_3_ receptors at the spinal cord level and via H_2_ receptors at the supraspinal level.

Importantly, the observations observed with H_3_ receptor‐KO mice are consistent with a pharmacological study using a preclinical model of neuropathic pain induced by chronic constriction injury of the sciatic nerve. Here, Popiolek‐Barczyk et al. (2018) showed that blockade of H_3_ receptors by a selective antagonist (E‐162) significantly enhanced morphine antinociception assessed with both mechanical and cold stimuli. Pharmacological analysis of these effects revealed an additive effect. Interestingly, Popiolek‐Barczyk et al. (2018) also showed that TR‐7, a selective H_4_ receptor antagonist, significantly enhanced morphine antinociception in neuropathic pain. This latter study is the first demonstration of the involvement of H_4_ receptors in the regulation of morphine efficacy in chronic pain.

To the best of our knowledge, the literature does not provide evidence for the mechanisms underlying histamine and opioid system interactions, in relation to the modulation of morphine analgesic effects. Given that the analgesic effects produced by modulation of the activity of both the histamine and opioid systems could be associated with blocking SP release from peripheral nerve terminals (Barnes et al., 1986; Przewłocki & Przewłocka, 2001), it is possible that an interaction that would result in potentiation of analgesic efficacy of morphine may involve, together with other possible mechanisms, the inhibition of peripheral SP accumulation. Such an outcome may be useful for the management of neuropathic pain, particularly when peripheral administration of drugs is possible, thus affording reduction of the undesired secondary effects associated with opioid administration and peripheral mechanisms of action (e.g., constipation). However, centrally acting drugs administered by peripheral routes should be taken into consideration due to the potential serious interactions related to their pharmacodynamics and central mechanisms of action. For example, chlorpheniramine (a first‐generation H_1_ receptor antagonist) was reported to potentiate fentanyl‐induced sedation and respiratory depression after surgery (Anwari & Iqbal, 2003).

## 6 CONCLUSIONS AND FUTURE DIRECTIONS

Findings from the last two decades indicate that selective pharmacological antagonism of neurons expressing H_3_ receptors could provide important and promising therapeutic approaches for the control of mechanical and cold hypersensitivity in peripheral neuropathies (Table 1). The analgesic effectiveness of H_3_ receptor antagonists/inverse agonists was comparable to gabapentin and pregabalin, first‐line treatments for neuropathic pain. Importantly, multiple examples of behavioural, electrophysiological, and molecular evidence strongly support the rationale for this neuropathic pain strategy, particularly given their ability to modulate histamine levels as well as several neurotransmitters critical for chronic pain processing. Moreover, the recent registered approval of pitolisant (Wakix™), an antagonist/inverse agonist of H_3_ receptors, for the treatment of narcolepsy in patients, has opened the door for the potential use of H_3_ receptor ligands for other conditions, including chronic neuropathic pain. However, due to the wide presynaptic and postsynaptic distribution of H_3_ receptors throughout the CNS and PNS, more research is certainly needed to clarify the involvement of peripheral, spinal, and brain H_3_ receptors in various pain states, before determining their full potential in neuropathic pain.

Recent findings also suggest the use of centrally permeable H_2_ receptor antagonists as promising new drug candidates for the treatment of neuropathic pain, in view of their analgesic effects and metabolic stability. Interestingly, however, despite the discovery of the most recently discovered histamine receptor, the role of the H_4_ receptor in neuropathic pain transmission is still controversial after nearly 20 years, with apparent confounding effects of both agonists and antagonists on hypersensitivity associated with neuropathic pain. This may be due to biased signalling of histamine and H_4_ receptor agonist ligands and differential effects on multiple signalling pathways in central and peripheral parts of the sensory nervous system. Furthermore, the paucity of detailed mechanistic definitions of histamine‐mediated analgesia, and the additive effects with the opioid system, requires attention to provide a rationale to the field of histamine and development of neuropathic pain control therapeutics.

A better understanding of the interaction between histaminergic signalling pathway molecules (Figures 1, 2, 3) and histamine receptors may result in the identification of further novel pharmacological targets to improve neuropathic pain control. The literature available provides some evidence for potential pharmacological target molecules. One potential strategy exploits the role of Ca^2+^ channels in the regulation of cellular excitability associated with nociception (e.g., N‐type Ca^2+^ channels). Evidence has shown that Ca^2+^ channel blockers (e.g., http://www.guidetopharmacology.org/GRAC/LigandDisplayForward?ligandId=2536) offer interesting analgesic potential in treating neuropathic pain (Vanegas & Schaible, 2000). However, there is no evidence for the effect produced by a combination of Ca^2+^ channel blockers and histamine receptor ligands, and we propose that their interaction should be taken into consideration. Another potential target involves the contribution of the MAPK/ERK signalling pathway to the regulation of pain hypersensitivity. Recently, Sanna et al. (2015) showed that H_4_ receptor stimulation, which led to analgesic activity in neuropathic pain, was modulated by MAPK/ERK signalling in the neurons of the DRG, spinal cord, and sciatic nerve. While the MAPK/ERK signalling pathway regulates pain sensitivity and, for a while, has been considered as a target for the treatment of neuropathic pain (Ma & Quirion, 2005), further studies on the interaction between this pathway and H_4_ receptors may lead to the identification of more efficient therapeutic strategies to control neuropathic pain.

### 6.1 Nomenclature of targets and ligands

Key protein targets and ligands in this article are hyperlinked to corresponding entries in http://www.guidetopharmacology.org/, the common portal for data from the IUPHAR/BPS Guide to PHARMACOLOGY (Harding et al., 2018), and are permanently archived in the Concise Guide to PHARMACOLOGY 2017/18 (Alexander, Christopoulos et al., 2017; Alexander, Fabbro et al., 2017; Alexander, Peters et al., 2017; Alexander, Striessnig et al., 2017).

## CONFLICT OF INTEREST

The authors declare no conflicts of interest.
